# Bridging the Gap in Psychogenic Erectile Dysfunction: A Narrative Review of Underexplored Therapeutic Options

**DOI:** 10.3390/medicina62091751

**Published:** 2026-09-11

**Authors:** Marta Pezzoli, Elettra Fuligni, Mattia Lo Re, Andrea Cocci, Andrea Minervini, Damien Carnicelli

**Affiliations:** 1 Department of Urology, Azienda Ospedaliera Universitaria Careggi, University of Florence, 50134 Florence, Italy; elettra.fuligni@unifi.it (E.F.); mattia.lore@unifi.it (M.L.R.); andrea.cocci@unifi.it (A.C.); andrea.minervini@unifi.it (A.M.); 2 Department of Urology, Hospices Civils De Lyon, Hôpital Lyon Sud, 69495 Oullins-Pierre-Bénite, France; damien.carnicelli@chu-lyon.fr

**Keywords:** erectile dysfunction, psychogenic erectile dysfunction, intracavernosal injections, vacuum devices, penile prosthesis, acupuncture therapy, cognitive behavioral therapy, patient selection

## Abstract

*Background and Objectives:* Psychogenic erectile dysfunction (ED) is a distinct clinical condition characterized by predominant psychological or relational factors. Despite its prevalence, diagnostic criteria remain heterogeneous and are often based on exclusion of organic causes. Current guidelines recommend cognitive behavioral therapy (CBT), eventually combined with medical therapy such as phosphodiesterase type 5 inhibitors (PDE5i), as first-line treatment. However, evidence regarding alternative conservative and surgical therapies remains limited. This narrative review aims to evaluate the available evidence on these treatments in psychogenic ED. *Materials and Methods:* A comprehensive literature search was conducted in PubMed, Scopus, and MEDLINE using MeSH terms and free-text keywords related to psychogenic ED and therapeutic interventions. Eligible studies included those involving human subjects, published in English, and reporting outcomes specifically for psychogenic or non-organic ED. Reviews, case reports, and non-full-text articles were excluded. Study selection and data extraction were performed independently by two authors. Due to heterogeneity, a narrative synthesis was conducted. *Results:* Evidence on alternative therapies is limited and heterogeneous, with many studies predating the 2000s. Vacuum erection devices (VEDs) may improve outcomes, particularly when combined with psychotherapy. Constriction rings showed promising preliminary results. Acupuncture demonstrated inconsistent efficacy, although recent randomized trials suggest potential benefits. Intracavernosal injections (ICI) showed favorable outcomes, particularly in patients refractory to first-line therapies, with some reports of restored spontaneous erections. Penile prosthesis (PP) implantation remains underexplored in contemporary literature on psychogenic ED; however, recent evidence reports good satisfaction rates. *Conclusions:* Beyond CBT and PDE5i, evidence for alternative treatments in psychogenic ED is scarce and methodologically limited. Selected patients may benefit from adjunctive therapies, including VED, ICI, and acupuncture, while PP can be considered a definitive treatment option. Further well-designed studies are needed to clarify their role within a structured, evidence-based management approach.

## 1. Introduction

Psychogenic erectile dysfunction (ED) is a clinically distinct form of ED in which psychological, relational, or contextual factors predominate in the absence of clear organic pathology. Despite its prevalence, definitions remain heterogeneous, often relying on exclusion rather than structured positive criteria [1,2].

Current guidelines, including those from the European Association of Urology (EAU), recommend cognitive behavioral therapy (CBT) as the first-line treatment, ideally combined with phosphodiesterase type 5 inhibitors (PDE5i) for optimized outcomes [3]. While this approach is well established, guidance on alternative conservative or surgical therapies remains limited, particularly for patients who are unresponsive to conventional management. In this context, the role of interventions widely studied for organic ED—including intracavernosal injections (ICI), mechanical devices, and penile prostheses (PP)—as well as alternative therapies such as acupuncture, remains underexplored [4].

PP implantation is especially controversial in psychogenic ED. Evidence is scarce, satisfaction outcomes are inconsistently reported, and current guidelines offer little direction regarding patient selection, timing, or psychological prerequisites for surgery [4].

This narrative review aims to critically examine the available evidence for both conservative and surgical treatments in psychogenic ED, highlighting gaps in knowledge and clarifying their potential role in carefully selected patients.

## 2. Materials and Methods

This review was conceived as a structured narrative review to provide a comprehensive overview of the available evidence. A literature search was conducted in PubMed, Scopus, and MEDLINE databases in March 2026 using a combination of Medical Subject Headings (MeSH) terms and free-text keywords related to psychogenic or non-organic ED. The search strategy included terms for ED combined with psychogenic descriptors and a broad range of therapeutic interventions, including pharmacological treatments (ICI), mechanical devices (vacuum erection devices and constriction rings), surgical options (PP), complementary therapies (acupuncture), energy-based therapies (shock wave therapy), and regenerative therapies (platelet-rich plasma, stem cells). No date restrictions were applied.

Only studies published in English and involving human subjects were considered eligible. Reviews, case reports, commentaries, letters to the editor, conference abstracts, and articles without full-text availability or published in languages other than English were excluded. Only studies reporting a clearly defined diagnosis of psychogenic or non-organic ED for some or all included patients, and providing outcome data specifically stratified for this subgroup, were eligible for inclusion in the review.

Titles and abstracts were screened independently to assess relevance to the study objectives. Full-text articles were retrieved when abstracts did not provide sufficient information for eligibility assessment. Data extraction and study selection were performed independently by two authors (M.P. and E.F.), with any discrepancies resolved through discussion with senior authors.

### 2.1. Evidence Synthesis

#### 2.1.1. Vacuum Erection Device Therapy

Vacuum erection devices (VEDs) have been described as a therapeutic option for both organic and psychogenic ED in few studies published mainly in the 1990s, with heterogeneous results [5,6,7,8].

On one hand, Meinhardt et al. reported complete treatment failure in a small cohort of 9 patients with pure psychogenic ED after three weeks of home-based VED use [5]. Conversely, more favorable outcomes have been described in other studies. Segenreich et al. evaluated the use of a multimodal approach combining VED with rational psychotherapy in patients with psychogenic (*n* = 38), organic (*n* = 53), and mixed (*n* = 54) ED. In the psychogenic subgroup, outcomes were comparable to those observed in patients with organic ED in terms of achieving adequate erection and maximal penile rigidity (>80%). Furthermore, patients with psychogenic ED showed the highest rate of successful intercourse (94.7%) following therapy; among these, 44.7% were able to maintain satisfactory sexual activity without continued use of VED, while 50% remained dependent on its ongoing use [6]. Similarly, El-Bahrawy et al., in a small cohort of 5 patients with psychogenic ED, reported successful erection achievement in all cases, with 60% of patients regaining the ability to have intercourse without the need for VED after one month of treatment [7].

Subsequently, a randomized study published in 2003 investigated the role of VED as an adjunct to psychosexual couple therapy. Patients were randomized to a VED group (Group 1) or a control group (Group 2), with both groups undergoing at least three psychotherapy sessions. The initial successful response to therapy was observed in 84% of patients in Group 1 compared with 60% in Group 2. Moreover, in Group 1, all couples in whom the device was effectively used achieved moderate-to-high improvement at final analysis, whereas the success rate in the control group was 60% [8].

#### 2.1.2. Constriction Rings

A recent study published in 2026 reported preliminary results on the use of Tenuto Mini^®^ (MysteryVibe, London, UK), a vibrating constriction ring evaluated over an eight-week period in 30 patients with psychogenic ED. The device was well tolerated, with no reported adverse events. Treatment was associated with a significant improvement in erectile function, with mean International Index of Erectile Function–5 (IIEF-5) scores increasing from 14.44 to 22.31, and a mean Erectile Dysfunction Inventory of Treatment Satisfaction (EDITS) score of 78.57% [9].

#### 2.1.3. Acupuncture Therapy

Heterogeneous results have been presented by early studies exploring the potential role of acupuncture in the treatment of psychogenic ED [10,11,12,13,14].

Yaman et al. reported a favorable response in 20 out of 29 patients with psychogenic ED following acupuncture treatment, with a variable number of sessions (mean 13.2) [11] . Conversely, a pilot study conducted in 1999 using a standardized protocol of eight acupuncture sessions targeting the same predefined acupoints showed more limited efficacy. Based on patient diaries, only 2 out of 9 patients with psychogenic ED demonstrated an improvement in erectile function, while 3 out of 9 reported increased sexual activity; however, these findings were not confirmed by their partners [10] .

Comparative studies have yielded inconsistent results. In one study, acupuncture was associated with an improvement in erectile function; however, the difference was not statistically significant compared with the placebo group [12]. In contrast, a randomized placebo-controlled trial, in which sham acupuncture was performed at points typically used for headache treatment, demonstrated a significantly higher rate of satisfactory response in the treatment group (68.4%) compared with the placebo group (9%; *p* = 0.017) [13].

More recently, Wang et al. conducted a randomized sham-controlled trial in 2023, representing the most up-to-date evidence on this topic. In this study, 66 patients with psychogenic ED were randomized (1:1) to either an acupuncture group or a sham acupuncture group, both undergoing a six-week treatment protocol consisting of eighteen sessions. At the end of treatment, the acupuncture group showed significantly greater improvements in the IIEF-5, Erection Hardness Score (EHS), and Sexual Encounter Profile Question 3 (SEP-3), as well as significantly lower scores on the Self-Rating Anxiety Scale (SAS) and Self-Rating Depression Scale (SDS) compared with the sham group (*p* < 0.05). No significant difference was observed between groups in SEP-2 outcomes (*p* > 0.05). The authors acknowledged that sham acupuncture, performed using superficial needle insertion at non-acupoints without manipulation, may still elicit physiological effects, representing a potential limitation of the study [14].

#### 2.1.4. Intracavernosal Injections

Five studies investigating ICI met the inclusion criteria [15,16,17,18,19].

Among these, Willke et al. analyzed a subgroup of patients with psychogenic ED (32%) treated with alprostadil injections administered one to three times per week over an eighteen-month period. Patients with psychogenic ED exhibited lower baseline mental health scores compared with other etiological groups, but demonstrated a greater improvement at the end of treatment [15]. Similarly, Kunelius et al. evaluated 31 patients with psychogenic ED treated with alprostadil in a three-year follow-up study. This subgroup showed the highest rate of improvement in erectile function (54.2%) compared with patients with other causes of ED, as well as a lower dropout rate (31.3%) than patients with vasculogenic ED [16].

In a study specifically focused on psychogenic ED, Baum et al. randomized 50 patients to receive either standard sex therapy or treatment with low-dose prostaglandin E1 (PGE1; 2.5–5.0 μg) for up to twelve weeks. Patients treated with PGE1 showed slightly lower rates of satisfaction and improvement in achieving unaided erections compared with those undergoing sex therapy (69% vs. 75% and 47% vs. 58%, respectively), but a longer duration of erection (35 min vs. 10 min) [17].

In more recent years, limited evidence has explored the use of ICI in this context. In one study involving young men (18–39 years) without identifiable organic causes, ICI were proposed only in a minority of patients (4/33; 12.1%) who were refractory to PDE5i [18]. In another study, conducted in young men (20–45 years) from traditional Muslim communities presenting with unconsummated marriages due to psychogenic ED and failure of PDE5i therapy, a rapid therapeutic effect was observed. Following one to two ICI with alprostadil and papaverine, all patients (12/12) reported restoration of spontaneous erections without the need for ongoing treatment [19].

#### 2.1.5. Penile Prosthesis

PP implantation has been investigated in the context of psychogenic ED in a limited number of included studies [20,21,22,23].. Notably, some early reports evaluated devices that are no longer available on the market.

In one of the earliest studies, Small reported the implantation of Small-Carrion prostheses in patients with both organic (*n* = 791) and psychogenic ED (*n* = 109). High overall satisfaction rates were described, and the author noted that PP were becoming “increasingly accepted” in patients with psychogenic ED [20]. Schlamowitz et al. evaluated satisfaction outcomes following inflatable PP implantation in a small cohort of patients with psychogenic (*n* = 7) and organic ED (*n* = 10). Although all patients reported that they would undergo the procedure again, satisfaction rates regarding penile length, mood improvement, sexual relationships, and sense of wholeness were lower in patients with psychogenic ED compared with those with organic ED [21].

In a 2025 comparative study by De Rocco Ponce et al., postoperative satisfaction following inflatable PP implantation was assessed in 25 patients with predominantly psychogenic ED (case group) and 36 patients with predominantly organic ED (control group). The psychogenic group reported high satisfaction rates (96%), with improvements in confidence to initiate sexual activity, ability to achieve penetration, and overall sexual performance. Additionally, 88% of patients stated that they would choose to undergo the procedure again, and 80% of partners reported satisfaction. A perceived morphological change in penile appearance was reported by 76% of patients. Both groups completed the Quality of Life and Sexuality with Penile Prosthesis (QoLSPP) questionnaire. Statistically significant better outcomes were observed in the psychogenic group compared with the organic group in 8 out of 16 items, while the remaining items showed comparable results between groups. However, surgical complications were more frequent in the psychogenic group (16%) compared with the organic ED group (2.8%), highlighting the need for careful patient selection and risk assessment in this population, although the reasons underlying this finding remain unclear [22].

Another recent study by Sevinç et al. investigated PP implantation in a cohort of young patients, including 18 out of 64 individuals without identifiable organic causes of ED. However, satisfaction data were available for only three patients in this subgroup: two reported being very satisfied, while one reported low satisfaction [24].

#### 2.1.6. Other Treatment Modalities

No eligible studies investigating low-intensity shockwave therapy, platelet-rich plasma, or stem cell therapies for psychogenic ED met the inclusion criteria. Therefore, no data on these emerging therapies were available for analysis.

Detailed characteristics of the included studies, including study design, diagnostic criteria for psychogenic/non-organic ED, interventions, outcomes, adverse events, and major limitations, are summarized in Table 1.

## 3. Discussion

Psychogenic ED represents a distinct clinical entity in which psychological and interpersonal factors play a predominant role. According to the EAU guidelines, the cornerstone of management is CBT, ideally involving the partner and—when appropriate—combined with medical treatment to optimize outcomes [3]. However, while psychological interventions are clearly recommended, specific guidance regarding adjunctive medical therapies remains limited [3].

PDE5i have been extensively investigated in this population. Over time, multiple systematic reviews, including those by Schmidt et al. and Atallah et al., have consistently demonstrated that the combination of psychological interventions and PDE5i is effective, and this approach can now be considered a standardized strategy [25,26] . Beyond PDE5i, however, the literature becomes markedly sparse and heterogeneous, particularly with regard to alternative options such as ICI, VED, and PP implantation.

A notable finding of the present review is that a substantial proportion of the available evidence on these treatments derives from studies conducted prior to the 2000s. In subsequent years, the focus progressively shifted toward predominantly psychological strategies and oral treatments [26,27] , a transition that may have limited the exploration of adjunctive interventions that could still benefit selected patients. Interestingly, more recent contributions suggest a renewed interest in this field, highlighting the increasing clinical relevance of revisiting and broadening available treatment strategies [4,9,14,18,19,22].

A major barrier to advancing research in this area appears to be the lack of a standardized and universally accepted definition of psychogenic or non-organic ED. These terms are often used interchangeably, contributing to persistent conceptual ambiguity. Although a “positive” definition—based on the identification of predominant psychological or interpersonal factors—has been advocated [1,2], in both clinical practice and research, psychogenic ED is still most commonly defined by exclusion, after ruling out identifiable organic causes. Efforts have been made to move beyond the traditional dichotomy, with studies adopting the classification of “primary psychogenic” versus “primary organic” ED, as proposed by the EAU Guidelines [28]. Nevertheless, the distinction itself may become increasingly blurred as emerging neuroimaging evidence reveals measurable neural alterations in patients with psychogenic ED, including abnormalities in functional connectivity and brain regions involved in emotional regulation, reward processing, and sexual arousal [29,30] . These recent findings support the view of psychogenic ED as a biopsychosocial condition with identifiable neurobiological correlates rather than merely a diagnosis of exclusion, potentially opening new avenues for targeted therapeutic interventions.

Considerable variability in diagnostic approaches to psychogenic ED emerges across the included studies. Methods range from clinical history and the use of both validated and non-validated questionnaires to hormonal evaluation, ICI testing, penile Doppler ultrasound (PDU), and nocturnal penile tumescence and rigidity (NPTR) assessments, applied in various combinations [17,20,21,22]. More recently, diagnostic pathways have been proposed, including an algorithm by Santi et al., which integrates clinical evaluation, hormonal testing, and a two-step ICI protocol, demonstrating high sensitivity and specificity [31]. In parallel, a Delphi consensus process has sought to standardize diagnostic criteria, identifying preserved morning erections and adequate rigidity during masturbation as key clinical indicators, while normal hormonal profiles, a normal ICI response, and poor response to PDE5i support a non-organic aetiology. Within this framework, NPTR, ICI testing, and PDU may serve as complementary diagnostic tools [4]. Overall, the development of a shared and structured diagnostic pathway appears essential to improve consistency across studies and strengthen the robustness of future evidence.

The current literature on alternative treatments remains limited and is characterized by several methodological constraints, including small sample sizes, heterogeneous populations, variable treatment protocols, and inconsistent definitions of therapeutic success—often reflecting the historical lack of validated outcome measures. Notably, no eligible studies were identified investigating emerging therapies such as low-intensity shockwave therapy, platelet-rich plasma, or stem cell therapies specifically in psychogenic ED.

Regarding VED, studies including larger patient cohorts suggest that its use in combination with psychological interventions—such as rational psychotherapy or psychosexual couple therapy—may be effective. In these contexts, VED appears to provide an additive benefit, improving erectile function and facilitating successful intercourse without significant adverse effects [5,8].

Acupuncture has yielded heterogeneous results in the treatment of psychogenic ED. A recent randomized, sham-controlled trial by Wang et al. reported significant improvements in erectile function as well as in psychological parameters, as assessed by validated questionnaires. However, the authors acknowledged that the sham intervention may not have been entirely inert, thereby limiting the interpretability of the findings [14]. More broadly, the substantial variability observed across acupuncture protocols represents a significant methodological challenge. This heterogeneity provided the rationale for a Delphi consensus process, which generated recommendations regarding acupoint selection, treatment frequency, and co-interventions, while also highlighting growing clinical interest among acupuncture practitioners in this field [32].

ICI have shown generally favorable outcomes, particularly in patients refractory to first-line therapies. Despite heterogeneity in treatment protocols and outcome definitions, several studies have reported meaningful improvements in erectile function and, in some cases, restoration of spontaneous erections following short treatment courses. These findings suggest that ICI may serve not only as a symptomatic treatment but also as a potential facilitator of psychosexual recovery in selected patients [19].

One of the most intriguing aspects concerns the role of PP implantation. Historically, PP was more frequently reported in the context of psychogenic ED in studies from the 1980s and 1990s, before largely disappearing from the literature. More recent studies, however, have reintroduced this topic [22,23,24,33] . While earlier data suggested slightly lower satisfaction rates in psychogenic compared to organic ED [21], more recent evidence from De Rocco Ponce et al. indicates high satisfaction rates, in some domains even exceeding those observed in organic ED, when assessed using validated instruments such as the QoLSPP questionnaire [22].

The relative paucity of contemporary studies on PP in psychogenic ED may be partly explained by healthcare policy and reimbursement limitations. For example, Krughoff et al. reported that PP implantation for psychogenic ED was covered by only 12.5% of insurance plans in the United States [34]. Similarly, in many national healthcare systems, reimbursement could be restricted to cases with documented organic aetiology. These constraints likely reduce the number of procedures performed in this population and, consequently, limit the feasibility of conducting large-scale studies. Additionally, they may encourage the classification of some patients under an organic aetiology for reimbursement purposes, potentially leading to an underestimation of the true number of PP implantations performed for psychogenic ED.

A recent expert consensus has endorsed the use of PP implantation in non-organic ED, recommending a stepwise pathway in which all non-surgical options—including PDE5i and ICI—are exhausted before surgery. As the most structured treatment framework currently available, this approach contrasts with the non-hierarchical treatment model adopted by the 2021 EAU Guidelines [35] and reflects a more cautious attitude toward surgical intervention in non-organic ED, considering both the irreversible nature of the procedure and the psychological complexity of the condition. Moreover, the consensus emphasizes mandatory preoperative psychological counseling, a dedicated informed consent process, involvement of the partner in the decision-making process when appropriate, and structured long-term follow-up including ongoing psychological support [4].

Overall, patients with psychogenic ED represent a clinically and etiologically distinct population, characterized by specific psychological and relational determinants that differentiate them from organic forms of ED. This heterogeneity underscores the importance of understanding whether treatments extensively studied in organic ED can be effectively translated to this subgroup, and to what extent they may contribute to meaningful improvements in both functional outcomes and psychosocial well-being.

Beyond the limitations already discussed, this review has additional methodological constraints. As a narrative review restricted to English-language publications, publication bias and the exclusion of unpublished studies cannot be excluded. Moreover, concomitant psychological interventions and/or PDE5i therapy may have confounded treatment effects, making it difficult to isolate the independent contribution of the investigated interventions.

## 4. Conclusions

Psychogenic ED remains a distinct clinical entity requiring a comprehensive and multidisciplinary approach. While CBT and PDE5i represent well-established and recommended treatments, additional therapeutic options—including VED, ICI, acupuncture, and PP—have been only marginally investigated in this population since the early 2000s. Despite this limited evidence base, these approaches may still provide meaningful adjunctive benefits in selected patients, particularly when standard therapies are insufficient.

Overall, the available literature is scarce and highly heterogeneous, with significant variability in study design, patient selection, treatment protocols, and outcome definitions, limiting the strength and generalizability of current evidence.

### Future Directions

Future research should prioritize well-designed, adequately powered studies specifically focused on psychogenic ED, with particular attention to treatment efficacy and patient satisfaction outcomes, in order to better define the role of these adjunctive therapies within a structured and evidence-based management framework.

## Figures and Tables

**Table 1:** Characteristics and outcomes of included studies.

Author, Year	Study Design	Sample Size	Terminology Used	Definition and Diagnostic Criteria	Intervention	Comparator	Follow-Up	Main Findings	Adverse Events	Major Limitations
**Meinhardt et al., 1993 ** [5]	Prospective observational clinical series/home-use trial.	74 men with ED; 9 with psychogenic ED.	Psychogenic impotence/psychogenic erectile failure.	Etiology established after history, physical examination and hormonal screening; RigiScan during visual stimulation, intracavernosal testing, home nocturnal tumescence/rigidity, cavernosometry/cavernosography, color Doppler, arteriography, neurophysiologic tests and psychiatric interview were used when appropriate.	VED (Pos-T-Vac or equivalent) used at home for 3 weeks after an office training session.	Descriptive comparison with other etiologic ED subgroups; no untreated control.	3-week home trial.	All 9 psychogenic patients failed treatment. Success was defined as willingness to purchase and continue using the device, rather than a validated erectile-function or satisfaction endpoint.	Across the full cohort, 8 men reported excessive pain and 1 developed penile-shaft skin necrosis; subgroup-specific adverse events were not reported.	Very small psychogenic subgroup; highly negatively selected, treatment-refractory population; no control group; subjective/nonvalidated definition of success; psychogenic diagnostic threshold not specified.
**Segenreich et al., 1995 ** [6]	Prospective uncontrolled comparative cohort.	145 men: 38 psychogenic, 53 organic and 54 mixed ED.	Psychogenic ED.	Classification based on physical examination, medical and psychosexual histories, penile-brachial index, nocturnal penile tumescence, penile duplex ultrasonography, electroencephalography and hormonal status. No explicit psychogenic cutoff was provided.	VED plus rational psychotherapy involving the patient and partner; 8–10 procedures over 1–2 months.	Organic and mixed ED subgroups receiving the same combined treatment.	1 year.	At 1 year, 36/38 (94.7%) achieved successful intercourse: 17/38 (44.7%) without further device use and 19/38 (50.0%) with continued VED use. Maximal penile rigidity >80% increased from 22.4% to 84.6%.	Not reported.	No randomized or untreated comparator; combined intervention prevents attribution to VED or psychotherapy; diagnostic criteria were broad and not operationalized; outcomes were largely nonvalidated.
**El-Bahrawy et al., 1995 ** [7]	Prospective case series.	21 men with ED of mixed etiologies; 5 with psychogenic ED.	Psychogenic impotence.	Intact morning erections and loss of an otherwise normal erection immediately before penile introduction/penetration.	Eric Aid vacuum device used before intercourse; constriction ring limited to 30 min.	Other etiologic subgroups were described; no formal control group.	Up to 6 months.	All 5 psychogenic patients achieved an erection sufficient for intercourse; 3/5 (60%) resumed intercourse without the device approximately 1 month after starting treatment.	Across the full cohort, transient penile discomfort occurred in 13/21 (62%) and severe suction-related pain in 4/21 (19%); no ecchymosis or penile fibrosis was observed.	Only 5 psychogenic patients; mixed-etiology cohort; uncontrolled design; no validated satisfaction or erectile-function instrument.
**Wylie et al., 2003 ** [8]	Randomized controlled trial.	45 couples: 25 allocated to VED plus therapy and 20 to therapy alone.	Predominant psychogenic ED/psychological ED.	DSM-IV diagnosis of male ED with psychogenic or combined etiology. ED was judged psychogenic when morning erections, situational erections (masturbation or another partner), or an acute non-traumatic onset were present.	Relationship therapy plus modified Masters and Johnson psychosexual therapy; VED introduced at session 2 in the intervention group.	The same couple-based psychosexual therapy without early VED provision.	Primary assessment after at least 3 sessions; final assessment at completion/discharge after a variable course of therapy.	Initial improvement was reported in 21/25 (84%) with VED versus 12/20 (60%) with therapy alone. Among men who actually used the VED, all couples achieved moderate-to-good improvement at final analysis; control-group improvement was 60%.	No formal adverse-event reporting. Five men did not use the VED because of embarrassment, perceived mechanical nature, partner objection or discomfort.	Small and underpowered trial; primary between-group difference did not reach conventional significance; therapists and participants were unblinded; therapist-rated global outcome; heterogeneous psychotherapy and subsequent crossover/additional treatments.
**Rodríguez Martínez et al., 2026 ** [9]	Prospective single-arm observational study (preliminary report).	30 men with psychogenic ED.	Psychogenic ED (pED).	Psychological factors were considered the principal drivers of ED; participants met DSM-5 criteria for Erectile Disorder.	Tenuto Mini vibrating constriction ring used over 8 weeks.	None.	8 weeks.	Mean IIEF-5 increased from 14.44 ± 3.64 to 22.31 ± 3.87 (*p* < 0.001). Mean EDITS treatment-satisfaction score was 78.57% (range 61.36–89.93%).	No discomfort, pain, skin irritation or other complications were reported.	Small uncontrolled preliminary study; short follow-up; no assessment of durability after device withdrawal; manufacturer supplied devices and the principal author reported consultancy work for the manufacturer.
**Yaman et al., 1994 ** [11]	Prospective uncontrolled case series.	29 men with purely psychogenic/non-organic ED.	Purely psychogenic impotence/non-organic etiology.	Absence of detectable organic pathology after history and physical examination, neurologic testing, endocrine evaluation, papaverine testing, color Doppler, cavernosometry/cavernosography and confirmatory nocturnal penile tumescence; all underwent detailed psychiatric evaluation.	Acupuncture at predefined points; 10 sessions, extended to 20 if initial treatment failed.	None.	3–12 months (mean 8 months).	Treatment success, defined as at least 2 sexual activities per week, occurred in 20/29 (69%). Four initially successful patients later returned to their pretreatment status.	Premature ejaculation was observed in 2 of 9 unsuccessful patients; no other serious adverse effects were reported.	No control or blinding; small sample; nonvalidated and unusually defined success endpoint; relapse occurred; treatment schedule was variable.
**Kho et al., 1999 ** [10]	Prospective pilot single-arm study.	16 enrolled, 13 completed; 9 completers classified as psychogenic ED.	Psychogenic ED/ED without organic comorbidity.	DSM-III-R ED with failure in >50% of intercourse attempts, positive erectile response to intracavernosal papaverine/phentolamine, detailed sexual and medical evaluation and penile duplex. Patients without an identified organic contributor were classified as psychogenic.	Eight standardized electroacupuncture sessions over 4 weeks.	None.	12-week observation period, including a final interview 1 month after treatment.	In the psychogenic subgroup, 2/9 reported improved erection quality and 3/9 increased sexual activity; partner diaries did not consistently confirm patient-reported improvement. No hormonal changes were detected.	Not reported.	Very small pilot study; no control or blinding; mixed etiologies; 3/16 did not complete; subjective diary-based outcomes; limited partner confirmation.
**Aydin et al., 1997 ** [12]	Controlled comparative study.	60 men: acupuncture *n* = 15, hypnosis *n* = 16 and placebo controls *n* = 29.	Non-organic male sexual dysfunction.	Male sexual dysfunction with no detectable organic cause; diagnostic work-up was not detailed in the abstract.	Acupuncture or hypnotic suggestion.	Placebo treatment.	Not reported.	Reported improvement was 60% with acupuncture, 75% with hypnosis and 43–47% with placebo. Acupuncture was not statistically superior to placebo; hypnosis appeared to be the only intervention superior to placebo. Patient reports were checked by partner interview.	Not reported in the abstract.	Randomization, blinding, diagnostic criteria, follow-up duration, outcome definition and adverse-event ascertainment were insufficiently described.
**Engelhardt et al., 2003 ** [13]	Prospective randomized placebo-controlled pilot trial with crossover of placebo nonresponders.	21 recruited; 20 completed. Initial allocation: 10 treatment and 11 placebo; 10 placebo nonresponders crossed over.	Psychogenic ED.	Normal nocturnal erections on 3-night RigiScan (>70% rigidity), normal sexual hormone levels, absence of organic comorbidities and sufficient erectile response to 50 mg sildenafil.	ED-specific acupuncture, 5–20 sessions (mean 11), once or twice weekly.	Acupuncture at points conventionally used for headache; placebo nonresponders crossed over to active treatment.	Serial assessment after 5, 10 and 15 sessions; no post-treatment long-term follow-up.	Active acupuncture produced erections sufficient for intercourse without further therapy in 13/19 (68.4%); 4/19 (21.1%) improved but still required sildenafil. Placebo response was 1/11 (9%). Mean total IIEF increased from 43.7 to 62.	No acupuncture-related side effects were observed.	Small pilot trial; crossover complicates parallel-group interpretation; placebo acupuncture may not have been physiologically inert; variable number of sessions; no durability assessment.
**Wang et al., 2023 ** [14]	Randomized, participant- and assessor-blinded, sham-controlled trial.	66 randomized (33 per group); 64 completed week-10 assessment.	Psychogenic ED (pED).	ED is attributed to non-organic factors such as anxiety, depression or relationship discord. Eligibility included IIEF-5 8–21 and stable sexual activity; history, laboratory evaluation, PDE5i plus audiovisual stimulation and, where indicated, ICI with color Doppler were used to exclude organic ED.	Acupuncture 3 times weekly for 6 weeks (18 sessions).	Superficial sham needling at non-meridian/non-acupoints without manipulation.	6-week treatment plus 4-week follow-up (week 10).	Primary IIEF-5 improvement at week 6 was greater with acupuncture (+7.97 ± 2.66) than sham (+3.58 ± 2.53). Acupuncture also produced greater EHS and SEP-3 improvement and lower anxiety/depression scores; SEP-2 did not differ significantly.	Two patients had transient post-acupuncture vertigo and one had localized bruising resolving within 2 weeks; no serious adverse events.	Sham needling may have physiological effects; only 4 weeks of post-treatment follow-up; single-center and modest sample; prior acupuncture exposure could affect blinding; intensive 18-session protocol may limit generalizability.
**Willke et al., 1998 ** [15]	Multinational open-label Phase III cohort with prespecified/secondary quality-of-life subgroup analyses.	848 enrolled; 732 entered home self-injection; 32% had psychogenic ED (approximately 234 men).	Psychogenic cause of ED.	Etiologic category was recorded in the parent trial, but this report did not provide the operational criteria used to diagnose psychogenic ED.	Intracavernosal alprostadil self-injection 1–3 times per week for 6 months, with an optional 12-month extension.	No placebo; comparisons across etiologic and dose subgroups.	Up to 18 months.	Patients with psychogenic causes had lower baseline mental-health scores and generally showed the greatest quality-of-life gains. In the overall cohort, 88% of injections during the first 6 months enabled satisfactory sexual activity; psychogenic-specific satisfaction/resolution rates were not separately reported.	Overall, penile pain occurred at some point in 44% and caused discontinuation in 3%; subgroup-specific adverse events were not provided.	No control group; psychogenic diagnosis not defined; secondary subgroup analysis; substantial attrition over time and use of last-observation-carried-forward/weighting; satisfaction outcomes not separately reported for the psychogenic subgroup.
**Kunelius and Lukkarinen, 1999 ** [16]	Three-year observational follow-up study.	95 invited; 69 attended follow-up, including 31 with psychogenic ED.	Psychogenic etiology.	Etiology was assigned after sexual history, physical examination, laboratory testing, intracavernosal PGE1 testing and penile duplex Doppler; no explicit psychogenic threshold was reported.	Long-term intracavernosal PGE1 self-injection.	Vasculogenic and neurogenic etiologic subgroups.	3 years; mean treatment exposure 23.3 months.	Thirteen of 31 psychogenic patients (41.9%) reported improved spontaneous erections, representing 54.2% of all 24 patients who improved. The psychogenic dropout rate was 31.3%. Overall satisfaction with home erections using PGE1 was 67.3%.	Overall: small hematomas 10.1%, priapism 4.3%, ultrasound-detected fibrosis 5.8% and prolonged pain leading to discontinuation 7.2%; no systemic adverse effects.	Observational mixed-etiology cohort; 26/95 did not attend follow-up; psychogenic diagnostic criteria were not specified; most satisfaction and safety results were not stratified by etiology.
**Baum et al., 2000 ** [17]	Randomized pilot comparative trial.	50 men; 25 assigned to low-dose PGE1 and 25 to sex therapy.	Psychogenic ED/psychogenic impotence.	Preserved full rigid morning/nocturnal and masturbatory erections; confirmation by home RigiScan (≥70% rigidity at base and tip for >5 min on at least 1 of 2 nights) or Snap-Gauge testing, plus office PGE1 response and normal endocrine/glucose evaluation.	Low-dose intracavernosal PGE1 (2.5–5.0 micrograms), self-administered up to 3 times weekly for up to 12 weeks.	Weekly standard sex therapy for up to 12 weeks.	4-, 8- and 12-week visits; patients also predicted performance 6 months later, but no objective 6-month follow-up was reported.	Treatment satisfaction was 69% with PGE1 versus 75% with sex therapy. Improvement in unaided erections was 47% versus 58%, respectively. Mean reported erection duration was 35 versus 10 min.	No priapism. One patient reported penile pain and injection-site bleeding, and one episodic ecchymosis; pain also contributed to dropout.	Small pilot study; substantial attrition (52% PGE1 and 64% sex-therapy completion); unblinded and largely self-reported outcomes; short treatment period; sildenafil became available during recruitment and influenced dropout.
**Wiggins et al., 2019 ** [18]	Retrospective chart review with prospective follow-up questionnaire.	73 men aged <40 years without an identifiable organic cause; 33 completed follow-up. Four of 33 used ICI.	ED with no identifiable organic cause; suspected psychogenic inhibition.	No organic cause after detailed medical, sexual and psychosocial history, examination, IIEF, biothesiometry, morning testosterone and basic laboratory studies; penile duplex with vasoactive injection was performed in selected PDE5i failures.	Treatment algorithm beginning with nightly tadalafil and psychosexual therapy; refractory patients could receive ICI, VED, intraurethral therapy or prosthesis.	Baseline status; no parallel control.	Minimum 6 months.	Among 33 respondents, 58% were satisfied with current erectile function and the mean IIEF erectile-function score increased from 13.3 to 18.8. Four men (12.1%) used ICI, but ICI-specific satisfaction or ED-resolution outcomes were not reported.	Not reported.	Retrospective, uncontrolled and heterogeneous treatment exposure; only 33/73 completed follow-up; population was defined by absence of organic disease rather than a standardized psychogenic diagnosis; ICI was used in only four men.
**Lidawi et al., 2022 ** [19]	Retrospective/observational case series.	12 young men with unconsummated marriages and psychogenic ED after PDE5i failure.	Psychogenic ED secondary to sexual performance anxiety.	Young men without vascular risk factors, with full rigid morning/night erections and generally preserved libido/ejaculation, whose erection was lost before penetration. Diagnosis was based on history; no laboratory or vascular testing was undertaken.	One or two home ICIs of low-dose papaverine plus alprostadil, titrated if needed.	None.	Available for all patients; conception outcomes were observed for up to 24 months, but a standardized ED follow-up interval was not specified.	All 12 achieved successful penetrative intercourse after 1–2 injections and subsequently resumed spontaneous erections without further treatment. Eight couples (75%) conceived after a mean of 8 months.	No adverse effects or priapism were reported.	Very small uncontrolled series; diagnosis based on history without objective exclusion testing; culturally specific population; no validated satisfaction instrument; recurrence and durability require longer standardized follow-up.
**Small, 1984 ** [20]	Large retrospective single-surgeon clinical experience/narrative surgical series.	900 prosthesis recipients; 109 categorized as having psychological problems.	Psychogenic impotence/psychological problems.	Psychogenic cases were generally men with persistent severe impotence after failed sexual counseling and/or psychiatric therapy. Evaluation included medical/sexual history, physical and laboratory assessment and routine NPT monitoring in psychogenic cases; no formal diagnostic threshold was stated.	Small-Carrion semi-flexible PP.	Descriptive comparison with multiple organic etiologies.	Not clearly reported for the psychogenic subgroup; series covered approximately 10 years of experience.	The author described high overall clinical success and increasing acceptance of implantation in selected psychogenic cases, but did not report psychogenic-specific satisfaction, intercourse success or ED-resolution rates.	Overall complication rate approximately 8%; infection reportedly fell from 15% in the first 20 cases to approximately 0.5% with antibiotic protocols. Psychogenic-specific adverse events were not reported.	Historical single-surgeon narrative series; no standardized psychogenic definition, comparator or validated satisfaction assessment; no subgroup-specific outcomes or clearly defined follow-up.
**Schlamowitz et al., 1983 ** [21]	Pilot comparative postoperative follow-up study.	17 respondents: 7 psychogenic and 10 organic; 12 partners also responded.	Psychogenic impotence/psychogenically impotent.	Impotence was failure to initiate an erection sufficient for penetration in at least 50% of encounters for at least 1 year. Differential diagnosis used 3-night NPT plus physical, urologic and psychological evaluation. Psychogenic cases required independent psychiatrist and psychologist judgments, failure of sex therapy, psychological stability and acceptable operative risk.	Inflatable PP implantation.	Men with organic impotence secondary to Peyronie’s disease.	1–4 years after implantation.	All patients stated they would undergo implantation again, but psychogenic recipients reported lower satisfaction with penile length, mood improvement, sexual relationships and sense of wholeness than organic recipients. Overall sexual activity increased.	All 7 psychogenic patients versus 4/10 organic patients reported additional operations, commonly for fluid loss, inflation/deflation problems, bulging or discomfort.	Extremely small sample; only 17/44 consenting patients returned data (and 17/75 initially contacted); major response/selection bias; descriptive analyses; historical prosthesis technology and nonvalidated study-specific questionnaires.
**De Rocco Ponce et al., 2025 ** [22]	Retrospective single-center case–control study.	61 men: 25 mainly psychogenic ED cases and 36 mainly organic ED controls.	Mainly/predominantly psychogenic ED.	Diagnosis based on clinical presentation, assessment by an experienced sexologist and 3-night NPTR. At least one erection with ≥60% rigidity for ≥10 min was considered normal. Peyronie’s disease, hypogonadism and prior prosthesis were excluded.	Three-piece inflatable PP implantation.	Patients with mainly organic ED undergoing the same procedure.	At least 6 months.	In the psychogenic group, 96% reported improved erections, 92% greater confidence initiating sex, 92% successful penetration and 95% satisfactory sexual encounters; mean satisfaction was 8.71/10, 88% would choose surgery again and 80% of partners were satisfied. QoLSPP results favored psychogenic cases in 8/16 items.	The study summary reported surgical complications in 16% of psychogenic versus 2.8% of organic patients; prosthetic infection was reported in 4% of psychogenic patients.	Small retrospective sample; Spanish translation of QoLSPP was not formally validated; different surgeons and implant brands; classical psychogenic/organic dichotomy may oversimplify mixed etiologies; relatively short minimum follow-up and limited direct partner data.
**Sevinç et al., 2025 ** [24]	Retrospective single-surgeon cohort.	64 men aged <40 years; 18 (28.1%) had no identifiable organic etiology. Satisfaction data were available for only 3 men in this subgroup.	Idiopathic ED/no possible organic etiological factor (not explicitly psychogenic ED).	Idiopathic ED was assigned when history, physical examination, intracavernosal alprostadil stimulation and penile dynamic duplex ultrasound showed no possible organic cause. No formal psychological assessment was reported.	Predominantly three-piece inflatable PP implantation.	Patients with identified organic etiologies within the same young cohort.	Median 7 years overall (range 0.5–24 years).	Of the 3 idiopathic/no-organic-cause patients with satisfaction data, 2 were very satisfied and 1 reported low satisfaction. Overall, 92.8% of 28 respondents were satisfied or very satisfied, but subgroup-specific ED resolution could not be determined.	Overall, 3/64 (4.7%) required revision (2 mechanical failures, 1 impending erosion); no infections. Subgroup-specific complications were not reported.	Retrospective, no external control, satisfaction response in only 28/64 and only 3/18 idiopathic patients; idiopathic/no-organic-cause classification is not equivalent to a confirmed psychogenic diagnosis; no standardized psychosexual assessment.

Abbreviations: DSM, Diagnostic and Statistical Manual of Mental Disorders; ED, erectile dysfunction; EDITS, Erectile Dysfunction Inventory of Treatment Satisfaction; EHS, Erection Hardness Score; ICI, intracavernosal injection; IIEF, International Index of Erectile Function; NPT/NPTR, nocturnal penile tumescence (and rigidity); PBI, penile-brachial index; PDE5i, phosphodiesterase type 5 inhibitor; PGE1, prostaglandin E1; PP, penile prosthesis; SEP, Sexual Encounter Profile; VED, vacuum erection device.

## Data Availability

No new data were created or analyzed in this study. Data sharing is not applicable to this article.

## References

[1] Rosen R.C. (2001). Psychogenic erectile dysfunction: Classification and management. Urol. Clin. N. Am..

[2] Bodie J.A., Beeman W.W., Monga M. (2003). Psychogenic erectile dysfunction. Int. J. Psychiatry Med..

[3] Capogrosso P., Corona G., Dinkelman-Smit M., Falcone M., Gül M., Kadioğlu A., Minhas S., Boeri L., Salonia A., Cocci A. (2026). EAU Guidelines on Sexual and Reproductive Health.

[4] Cocci A., Pezzoli M., Pizziconi V., Bettocchi C., Salonia A., Minhas S., Boeri L., Capogrosso P., Cilesiz N.C., Gül M. (2026). Evaluating the appropriateness of penile prosthesis in non-organic erectile dysfunction and premature ejaculation: A Delphi consensus among penile implant surgeons. J. Sex. Med..

[5] Meinhardt W., Lycklama à Nijeholt A.A.B., Kropman R.F., Zwartendijk J. (1993). The negative pressure device for erectile disorders: When does it fail?. J. Urol..

[6] Segenreich E., Israilov S.R., Shmueli J., Servadio C. (1995). Vacuum therapy combined with psychotherapy for management of severe erectile dysfunction. Eur. Urol..

[7] El-Bahrawy M., El-Baz M.A., Emam A., El-Magd M.A. (1995). Noninvasive vacuum constriction device in the management of erectile dysfunction. Int. Urol. Nephrol..

[8] Wylie K.R., Jones R.H., Walters S. (2003). The potential benefit of vacuum devices augmenting psychosexual therapy for erectile dysfunction: A randomized controlled trial. J. Sex Marital Ther..

[9] Rodríguez Martínez J.E., Sosa Cabrera M., Dadian R., Suarez Beltrán V., Sanchez Llobregat L. (2026). Efficacy of a vibrating constriction ring for psychogenic erectile dysfunction: Preliminary results. Int. J. Impot. Res..

[10] Kho H.G., Sweep C.G.J., Chen X., Rabsztyn P.R.I., Meuleman E.J.H. (1999). The use of acupuncture in the treatment of erectile dysfunction. Int. J. Impot. Res..

[11] Yaman L.S., Kiliç S., Sarica K., Bayar M., Saygin B. (1994). The place of acupuncture in the management of psychogenic impotence. Eur. Urol..

[12] Aydin S., Ercan M., Çaşkurlu T., Taşçi A.I., Karaman I., Odabaş Ö., Yilmaz Y., Ağargün M.Y., Kara H., Sevin G. (1997). Acupuncture and hypnotic suggestions in the treatment of non-organic male sexual dysfunction. Scand. J. Urol. Nephrol..

[13] Engelhardt P.F., Daha L.K., Zils T., Simak R., König K., Pflüger H. (2003). Acupuncture in the treatment of psychogenic erectile dysfunction: First results of a prospective randomized placebo-controlled study. Int. J. Impot. Res..

[14] Wang H., Lei X., Ma D., Zhao Z., Wang A., Du G., Zhang J., Wang F., Guo J. (2023). Efficacy of acupuncture for psychogenic erectile dysfunction: A randomized, sham-controlled trial. Basic Clin. Androl..

[15] Willke R.J., Yen W., Parkerson G.R., Linet O.I., Erder M.H., Glick H.A. (1998). Quality of life effects of alprostadil therapy for erectile dysfunction: Results of a trial in Europe and South Africa. Int. J. Impot. Res..

[16] Kunelius P., Lukkarinen O. (1999). Intracavernous self-injection of prostaglandin E1 in the treatment of erectile dysfunction. Int. J. Impot. Res..

[17] Baum N., Randrup E., Junot D., Hass S. (2000). Prostaglandin E1 versus sex therapy in the management of psychogenic erectile dysfunction. Int. J. Impot. Res..

[18] Wiggins A., Tsambarlis P.N., Abdelsayed G., Levine L.A. (2019). A treatment algorithm for healthy young men with erectile dysfunction. BJU Int..

[19] Lidawi G., Asali M., Majdoub M., Rub R. (2022). Short-term intracavernous self-injection treatment of psychogenic erectile dysfunction secondary to sexual performance anxiety in unconsummated marriages. Int. J. Impot. Res..

[20] Small M.P. (1984). Semi-flexible penile prosthesis for organic and psychogenic impotence: Emphasis on preoperative, intraoperative and postoperative considerations. World J. Urol..

[21] Schlamowitz K.E., Beutler L.E., Scott F.B., Karacan I., Ware C. (1983). Reactions to the implantation of an inflatable penile prosthesis among psychogenically and organically impotent men. J. Urol..

[22] De Rocco Ponce M., Silva Garretón A., Sousa Iglesias Á., Dumas Castro S., Contreras Garcia R., Malca Caballero L., Rene Sanchez Curbelo J., Vantman Luft D., Castañé E.R., Rajmil O. (2025). Patient satisfaction and outcomes of penile prosthesis implantation in psychogenic and organic erectile dysfunction: A comparative study. J. Clin. Med..

[23] Sevinç A.H., Şenel S., Teke İ., Dursun M., Kadıoğlu A. (2025). Penile prosthesis implantation in patients under 40: Do etiological factors differ?. J. Sex. Med..

[24] Sevinç A.H., Şenel S., Teke İ., Gürcan M., Ergül R.B., Dursun M., Kadıoğlu A. (2025). Outcomes of penile prosthesis implantation in patients under 40: Insights and efficacy. Int. J. Urol..

[25] Schmidt H.M., Munder T., Gerger H., Frühauf S., Barth J. (2014). Combination of psychological intervention and phosphodiesterase-5 inhibitors for erectile dysfunction: A narrative review and meta-analysis. J. Sex. Med..

[26] Atallah S., Haydar A., Jabbour T., Kfoury P., Sader G. (2021). The effectiveness of psychological interventions alone, or in combination with phosphodiesterase-5 inhibitors, for the treatment of erectile dysfunction:A systematic review. Arab J. Urol..

[27] Dewitte M., Bettocchi C., Carvalho J., Corona G., Flink I., Limoncin E., Pascoal P., Reisman Y., Van Lankveld J. (2021). A psychosocial approach to erectile dysfunction: Position statements from the European Society of Sexual Medicine (ESSM). Sex. Med..

[28] Pozzi E., Fallara G., Capogrosso P., Boeri L., Belladelli F., Corsini C., Costa A., Candela L., Cignoli D., Cazzaniga W. (2022). Primary organic versus primary psychogenic erectile dysfunction: Findings from a real-life cross-sectional study. Andrology.

[29] Tian Z., Ma Z., Dou B., Huang X., Li G., Chang D., Yin T., Zhang P. (2025). Altered gray matter morphometry in psychogenic erectile dysfunction patients: A surface-based morphometry study. Sci. Rep..

[30] Liu X., Niu P., Yang Z., Xu Y., Liu T., Liu S., Chen Y., Chen J. (2025). Altered effective connectivity in reward-related circuits of psychogenic erectile dysfunction: Insights from spectral dynamic causal modeling. Eur. J. Neurosci..

[31] Santi D., Spaggiari G., Simoni M., Granata A.R.M. (2022). Accurate and time-saving, two-step intracavernosal injection procedure to diagnose psychological erectile dysfunction. Andrology.

[32] Dahle M.L., Alræk T., Musial F. (2024). An acupuncture protocol for the treatment of erectile dysfunction: A Delphi process. Complement. Med. Res..

[33] Sarna S., Reid T., Di Giovanni A., Holden F., Li C.Y., Lee W.G., Christopher A., Ralph D., Sangster P. (2025). Implantation of penile prosthesis in men 35 years and younger: A case series. J. Sex. Med..

[34] Krughoff K., Munarriz R.M., Gross M.S. (2021). An assessment of current penile prosthesis reimbursement guidelines for insurance plans nationwide. Int. J. Impot. Res..

[35] Salonia A., Bettocchi C., Boeri L., Capogrosso P., Carvalho J., Cilesiz N.C., Cocci A., Corona G., Dimitropoulos K., Gül M. (2021). European Association of Urology guidelines on sexual and reproductive health—2021 update: Male sexual dysfunction. Eur. Urol..

